# Quality of life of deaf and hard of hearing students in Ibadan metropolis, Nigeria

**DOI:** 10.1371/journal.pone.0190130

**Published:** 2018-01-02

**Authors:** Mofadeke T. Jaiyeola, Adebolajo A. Adeyemo

**Affiliations:** Institute of Child Health, College of Medicine, University of Ibadan, Ibadan, Nigeria; TNO, NETHERLANDS

## Abstract

Quality of Life encompasses an individual’s well-being and health, social participation and satisfaction with functional daily living. Disabilities such as deafness can impact on the quality of life with spatial variance to the environment. Deafness causes communicative problems with significant consequences in cognitive, social, and emotional well-being of affected individuals. However, information relating to the quality of life of deaf and hard of hearing individuals, especially students in developing countries like Nigeria, which could be used to design special health-related interventions is sparse. This study examined the quality of life of deaf and hard of hearing students in Ibadan metropolis, Nigeria. One hundred and ten deaf and hard of hearing students participated in this cross-sectional study. Participants were drawn from all four secondary schools for the Deaf in Ibadan metropolis. The 26 item Brief version of the WHO Quality of Life questionnaire was used for data collection. The data was analyzed using descriptive and inferential statistics at statistical significance of p<0.05. Majority (57.8%) of the deaf and hard of hearing students had poor quality of life. Attending the special school for the Deaf, upper socio-economic status and age (≥17years) are significantly associated with better quality of life. However, gender and age at onset of hearing loss had no significant influence on the quality of life. The Deaf community available in the special school appeared to protect against stigma and discrimination, while also promoting social interactions between deaf and hard of hearing individuals.

## Introduction

Hearing loss is an invisible health condition with important implications on the individual’s quality of life. Approximately 5.3% of the world’s population (360 million people) suffers from disabling hearing loss; majority of individuals with disabling hearing loss live either in low or middle-income countries [1]. In Sub-Saharan Africa, the prevalence of disabling hearing loss among children is 1.9%, while in adults male and females it is 7.4% and 5.5% respectively [2].

Hearing loss, though substantially underestimated and under-treated [3] is often a life-long disability that can cause profound damage to the development of speech, language, and cognitive skills in children depending on the severity and affected speech frequencies [4]. Thus, hearing loss alters progress in school and subsequent ability to obtain and keep employment [4]. Since hearing loss deters the acquisition of language in addition to speech and cognitive skills in children, this disability pose a major difficulty during childhood [5]. There are differing opinions on which type of school system–the inclusion and the exclusion systems–is more suitable for better cognitive development of a Deaf and Hard of Hearing (DHH) child. In the exclusion system, DHH students are taught in special schools and classrooms using special techniques and equipment by specialist personnel [6]. These schools cater for the Deaf community only, providing a wide range of special services such as sign language teachers, counselors, psychologists, and audiologists but they are typically small, accommodating only about 150–200 students [7]. Communication at these schools is usually via sign language, both formally in the classroom and informally among students enabling them to build friendships, self-esteem, self-confidence and social competence. These schools also offer relatively more opportunities for DHH students to take up leadership positions than in mainstream schools [8]

In the inclusion system also known as mainstreaming, classes are combined with special education services in a regular system. This system allows for strategized, continued and planned interactions between students [9]. Mainstreaming has two possible tracks: (a) Total Mainstreaming: whereby a DHH student attends a regular school with typical hearing students. Special services, such as interpreters, note takers or speech therapy are often needed. (b) Partial Mainstreaming: whereby a DHH student attends a regular school with typical hearing students but receives all or most of the classes in a special resource room. Mainstream schools provide opportunities for interaction between DHH students and their colleagues with typical level of hearing; this is thought to be beneficial to the DHH students [10, 11]. The decision to integrate DHH students in mainstream schools is often based on possible cognitive gains, though careful consideration of the social, psychological and academic consequences on the DHH students is required [7, 12] due to reports of difficult relationships between the two groups of students in the mainstream schools [8, 13].

A profound hearing loss is a major disability which affects all aspects of life and has varying effects on different individuals [14]. The variation in the effects of hearing loss can be attributed to certain factors such as environment, educational level and socioeconomic status. The Quality of Life (QoL) concept is important to understand children and youths with hearing loss because of the importance of communication and social participation in daily life [15]. Majority of DHH children have parents with typical hearing levels and about 80% of these parents are unable to effectively communicate and engage in deep communication with their DHH offspring [16]. Therefore, a DHH child born into a family with typical hearing may be unable to participate adequately in family conversations with subsequent significant adverse impact on the child [17]; the adverse effects experienced by the DHH child or adolescent include feelings of excommunication during family gatherings because of communication difficulties and failure of family members with typical hearing to realize the isolation of the DHH child [7]. However, for the approximately 12% of DHH children born to DHH parents there is a different scenario [16]: their natural language is sign language, because parents communicate with them from birth with sign language [18]. Such DHH children often have better social and emotional adjustment than DHH children born to parents with typical hearing [19]. Moreover, in families with more than one DHH child, sign language communication between the DHH siblings improves acceptance and encourages healthy cognitive and social development irrespective of the hearing status of the parents [13].

The American Sign Language (ASL) is the most commonly used method of communication for DHH individuals in Nigeria today [20]. In many developing countries like Nigeria, efforts made to integrate DHH individuals into the society are usually non-existent or feeble at best. This may affect the QoL of DHH individuals because social interaction is an important factor in predicting the physical health and well-being of an individual [21]. Deafness is associated with important adverse effects on the QoL, greater dysfunction being associated with more severe hearing loss [22]; moreover, the disrupted interpersonal communication could initiate social and behavioral problems in DHH young people [23]. Unlike in Europe and North America, the experiences of DHH young people and the effects of the disability on their QoL has not been fully studied in developing countries yet. Nigeria, like many developing countries has sparse data on the QoL of DHH young people. Such data is required for population needs assessment, intervention programs design, evaluation and educational placement. This study sought to determine: (1) the QoL of DHH students (2) impact of school system on QoL and (3) factors affecting the QoL of DHH students in Ibadan metropolis, Southwest Nigeria.

## Methods

### Study design

The study adopted a descriptive cross-sectional design that assessed the QoL of DHH students using a quantitative method of data collection.

### Study setting

The study was conducted in Ibadan, the capital of Oyo State, South-west Nigeria. Ibadan is located 78 miles inland from Lagos—the economic hub of Nigeria—and is a prominent link between the coastal region and the northern region of the country [24]. The population of the city is approximately 3,800,000 according to 2006 census estimates [25]. Ibadan metropolis is made up of 11 local government areas and has four secondary schools for the Deaf. Two of the schools are Total Mainstream Schools (TMS) where DHH students are in the same class with typical hearing students, and provision of interpreter services during lecture periods; one was a Partial Mainstream School (PMS) where DHH students are in the same school with typical hearing students but in separate classes, interpreter services are available for all lecture periods. The last school is a special school for DHH students only.

### Study population and sampling procedure

All the DHH students in senior secondary class 1 and class 2 in all the four schools for the Deaf in Ibadan who met the inclusion criteria: (1) deaf and hard of hearing, (2) use of sign language as the primary language and, (3) volunteered to be part of the study were surveyed. The DHH population in this study was defined as students enrolled in secondary schools for the Deaf in Ibadan, Nigeria with sufficiently non-functional hearing, thus, requiring use of sign language as the primary language of communication. This category of students had no other co-morbidities including self-identified intellectual impairment, learning disabilities or other types of physical disabilities. None of the students had a cochlear implant. Students were enrolled in schools for the Deaf after submission of audiogram test result from recognized government hospitals. A total of 110 students were sampled, the interviewer and a sign language interpreter were present throughout the interview period to guide the students and interpret difficult words throughout the questionnaire filling process. Eight questionnaires were not completely filled and were thus exempted from the analysis. All results shown are for 102 respondents.

Ethical approval was obtained from the University of Ibadan/University College Hospital Ibadan Institutional Review Board (IRB). The IRB approved the use of verbal assent from the students after informed consent had been given by the school heads, the consent of the school heads was deemed in place of parental consent. After consent was obtained from the appropriate authority of each of the schools, a sign language interpreter communicated the goals of the study and other contents of the approved informed consent documents to the students. After it was established that the students understood the study, they were asked to indicate their willingness to participate in the study by show of hands. Only students who volunteered to be part of the study were subsequently enrolled.

### Data collection instruments

The data was collected with an interviewer-assisted, semi-structured questionnaire examining socio-demographic characteristics, information on hearing loss and, the 26 item Brief version of the WHO Quality of Life questionnaire (WHOQOL-BREF) was used to measure the QoL of the students [26]. The WHOQOL-BREF comprises questions about the respondents QoL, health, other segments of their lives, and their experiences in the four weeks prior to the study. All the items in WHOQOL-BREF have five options each ranging from the highest to the lowest score (5–1). Total obtainable score on the WHOQOL -BREF ranged from 26–130; the median score ‘78’ was used as the cutoff point. QoL score ≤78 was classified as poor while QoL score >78 was regarded as good [27]. The WHOQOL-BREF is a versatile instrument and has been widely used in Nigeria to study QoL [28–30]. Socio-economic classification was adapted from the model designed by Oyedeji G. [31]. Briefly, this involved awarding values to the level of education and occupation of the respondent’s parents or guardian. Mean score of the values for both parents were computed and this denoted the socio-class each participant was placed.

### Validity and reliability of the instruments

The suitability of the tool to the study population was pretested in another town among DHH students with similar characteristics to the study group. Reliability of the WHOQOL-BREF questionnaire for the population studied was checked and Cronbach’s alpha coefficient was 0.85.

### Method of data analysis

IBM SPSS Statistics version 20.0 (Armonk, New York, USA) was used for the data analysis. The proportion of students in the defined categories was analyzed using ANOVA, chi-square and multiple regression tests. The level of statistical significance was set at 5%.

## Result

One hundred and two (102) respondents completed all measures of the study (S1 Table). The DHH students were between ages 12 and 31 years (45 males and 57 females with a mean age of 17.8±2.3years) (Table 1). There were 29 (28.4%) participants from the special school, 31 (30.4%) and 42 (41.2%) from the partial mainstream and total mainstream schools respectively. The middle social class constituted 43.1% of the study population while 28.4% were from the upper social class (Table 1) (S2 Table).

**Table 1:** Socio-demographic characteristics.

Variables	TMS N = 42		PMS N = 31		Special School N = 29		Total N = 102	
	Frequency	%	Frequency	%	Frequency	%	Frequency	%
**AGE(Years)**								
12–15	12	28.6	1	3.2	2	6.9	15	14.7
16–19	23	54.8	23	74.2	17	58.6	63	61.8
20–23	6	14.3	6	19.4	8	27.6	20	19.6
24–27	1	2.4	1	3.2	0	0	2	
28–31	0	0	0	0	2	6.9	2	1
**GENDER**								
Male	23	54.8	10	32.3	12	41.4	45	44.1
Female	19	45.2	21	67.7	17	58.6	57	55.9
**SOCIAL CLASS**								
Upper	2	4.0	6	19.4	21	72.4	29	28.4
Middle	20	47.6	18	58.1	6	20.7	44	43.1
Lower	20	47.6	7	22.5	2	6.9	29	28.4

### Disparities in the quality of life profile of respondents

The QoL profile of respondents based on scores from the WHOQOL-BREF are shown in Table 2. Majority of the study participants (57.8%) reported poor QoL, while 42.2% reported good QoL. The mean QoL scores for each of the four domains examined were higher in the special school respondents with statistically significant differences in social relationship and environment domains (S3 Table).

**Table 2:** Quality of life profile of respondents.

QoL Domains	Type of School				
	Special School	PMS	TMS	F-Test (ANOVA)	P-value
**Physical Health**					
MEAN±SD	23.44 ± 3.87	21.58 ± 3.90	21.79 ±4.84	1.733	0.182
**Psychological Health**					
MEAN±SD	19.01 ± 4.16	16.81 ± 3.31	17.67 ± 4.13	2.64	0.07
**Social Relationship**					
MEAN±SD	9.71 ± 2.28	8.87 ± 1.71	8.54 ± 1.88	3.182	0.046
**Environment**					
MEAN±SD	25.21 ± 4.69	21.87 ± 3.91	21.52 ± 4.46	6.863	0.002
**Total QoL Score**					
MEAN±SD	83.83 ± 10.79	74.19± 10.62	74.50 ± 11.93	7.370	0.001

Respondents’ QoL varied significantly across the different schools and socioeconomic classes but not between gender and age at onset of hearing loss (Table 3). A significantly higher proportion of participants from special school (48.8%) had good QoL compared to the partial (20.9%) and total mainstream schools (30.2%). DHH students aged less than 17 years had predominantly poor QoL (84.6%) while majority of those aged 17 years or more had good QoL (51.3%). Similarly, in the comparison of the socioeconomic status, a significantly higher proportion of participants from the upper socioeconomic class (46.5%) had good QoL compared to the middle (39.5%) and lower classes (14%). More females (53.5%) than males (46.5%) had a good QoL. More participants (76.3%) who had pre-lingual deafness (aged 0–4 years when hearing loss occurred) had a poor QoL compared to those who had post-lingual deafness (aged ≥ 5 years when hearing loss occurred) (Table 3).

**Table 3:** Quality of life status according to the independent variables examined.

	Poor QOL N = 59 [57.8%]	Good QOL N = 43 [42.2%]	Total N = 102 [100%]	Odds ratio	P-value
**Variable**					
School Type					
**Special**	8 [13.6%]	21[48.8%]	29 [28.4%]	15.24	<0.001
**PMS**	22[37.3%]	9 [20.9%]	31 [31.4%]		
**TMS**	29 [49.2%]	13 [30.2%]	42 [41.2%]		
Social class					
**Upper**	9 [15.3%]	20 [46.5%]	29 [28.4%]	14.25	<0.001
**Middle**	27[45.8%]	17 [39.5%]	44 [43.1%]		
**Lower**	23 [39.0%]	6 [14.0%]	29 [28.4%]		
Age (Years)					
**<17**	22 [84.6%]	4[15.4]	26[25.5%]	10.25	0.001
**≥17**	37[48.7%]	39[51.3%]	76[74.5%]		
Gender					
**Male**	25 [42.4]	20 [46.5%]	45 [44.1]	0.173	0.678
**Female**	34 [57.6%]	23 [40.4%]	57 [55.9]		
Age at onset of hearing loss (years)					
**0–4**	45 [76.3%]	34 [79.1%]	79 [77.5%]	0.112	0.738
**≥5**	14 [23.7%]	9 [20.9%]	23 [22.5%]		

### Predictors of good quality of life

Multiple regression analysis was done to investigate how the factors listed in Table 3 above predict a good QoL among the respondents (Table 4). Students in PMS were 5 times less likely (adjusted OR: 4.74 95%CI: 0.053–0.832) to have a good QoL compared to those in special school, while students from the upper social class had 6 times higher odds of a good QoL (adjusted OR: 5.95 95%CI: 1.257–28.14) compared to those from the lower socioeconomic classes. Age was a significant predictor of good QoL, students ≥17 years have 7 times higher odds of a good QoL compared to those <17 years of age.

**Table 4:** Adjusted odds ratio estimate for good QoL.

Factors	Significance	Adjusted odds ratio	95% CI
**Type of school**			
Special		1	
PMS	0.026	0.211	0.053–0.832
TMS	0.121	0.308	0.069–1.363
**Gender**			
Male	0.283	1.726	0.637–4.676
Female		1	
**Social class**			
Upper	0.025	5.948	1.257–28.144
Middle	0.071	3.065	0.908–10.347
Lower		1	
**Age at onset of hearing loss (years)**			
0–4	0.147	2.526	0.722–8.835
≥5		1	
**Age (years)**			
<17		1	
≥17	0.005	6.564	1.789–24.081

Model chi-square value: 32.755; Nagelkerke R-square: 0.37; Sig: 0.000

## Discussion

Perceived QoL is defined as an individual’s perception of their position in life in the context of the culture and value systems in which they live and in relation to their goals, standards, expectations and concerns [32]. Every individual–including DHH young people–perceives their QoL uniquely. While many reasons may be adduced for an individual’s dissatisfaction with their QoL, any form of disability is a threat to existence and has great impact upon the QoL of an individual [33]. Hearing loss can have a detrimental effect on the QoL of individuals in all domains [34]; this has led to a growing clinical interest in the effects of hearing loss in young people especially in developing countries.

The poor QoL seen in the majority of DHH students in this study conforms to reports from other countries which suggests that DHH young people have poor QoL; those studies showed that hearing loss had a significant detrimental impact upon overall QoL [34–36]. It has been suggested that the differences in QoL between people with typical hearing levels and those with hearing loss may be similar to the reported differences in QoL between people with chronic illness (such as sickle cell disease, obesity) and those who are otherwise healthy [37–39]. A study in rural USA showed that general life satisfaction in DHH youth was found to be poor in the domains of self, family, friends, and living environment [15]. Another study in Austria also reported that deafness has debilitating effect on the QoL of an individual [33].

Respondents in this study were from both the inclusive and special schools; the choice of either segregated or integrated placements for DHH students in Nigeria gives an option in choosing from different complementary forms of social experience that contributes uniquely to their overall adjustment [40]. Three of the schools for the study are government owned (public) non-residential schools while the special school is a private residential school.

A significant difference in the QoL of DHH students in the different school settings was observed; students from the special school had better QoL score than other schools. The significant influence of the school type on perceived QoL of DHH young people has been reported earlier [30]. Nevertheless, some other authors did not find any significant effect of school type on QoL of DHH young people [41]. Although DHH students could benefit from both the special and mainstream schools, DHH students in special schools tend to have good QoL because students in this type of school learn and interact within an environment that does not regard deafness as a deficiency rather, fostering acceptance of deafness [30]. Moreover, DHH students in special schools do not encounter negative attitudes from students with typical hearing which may enhance their QoL unlike their colleagues in mainstream schools who are likely to face criticism and discrimination from students with typical hearing [10, 11]. It has been demonstrated that deaf-specific programs promote more successful socio-emotional growth compared to mainstream schools [42]. Nonetheless, DHH students in mainstream schools have the opportunity to interact with students with typical hearing; this may be a great benefit since the DHH students have an opportunity to learn how to function in the ordinary society [42]. Another possible explanation for a better QoL among the special school students could be that most public schools in developing countries including Nigeria tend to be overcrowded. This implies that the overburdened teaching personnel pay minimal attention to individual students unlike private schools which tend to be exclusive and the personnel pays greater attention to each student. Furthermore, there may be lack of instructional materials and skilled sign language personnel in the public schools [43]. In addition, students in special school showed significantly higher social relationship than those in other schools, this result suggest that separating DHH students from students with typical hearing produces a significant effect on their social relationships.

In this study, the majority of the students from the upper class (72%) attend the special school; this may be an additional explanation for the better QoL seen in the students in the special schools compared to their counterparts in other schools; improved socio-economic status has been reported to have a positive bearing on all aspects of an individual’s life [44]. Several studies from Europe and US have shown that the association between socioeconomic status and health follows a common pattern in which individuals in the lower socioeconomic status have a poorer state of health [45, 46]. Similar results obtained in other countries demonstrates that this association is true in spite of differences in cultural backgrounds or economic growth [44]. The association between socioeconomic status and health seen in studies from several countries establishes socioeconomic status as a key factor that influences QoL for children, youths and families; affecting human activities in many ways, including development across the life span, psychological health and physical health [47]. Moreover, individuals from lower social classes are more vulnerable as they are likely to be exposed to more stressful experiences than upper class individuals. These stressful events may have comparably more severe impact on their emotional functions than on the individuals from the upper class [48].

This study showed that though majority of the study participants (77.5%) became DHH early in life, the age at onset of hearing loss did not significantly impact QoL. Despite the lack of statistical significance, 76.3% of students with poor QoL became DHH early in life. Another Nigerian study reported that the deleterious effects of hearing loss which occurred very early in life are usually more grievous than hearing loss after acquisition of language [34]. Our results have shown that there is no difference in the QoL of deaf and hard of hearing students according to gender, similar to other studies already published which showed that gender was not associated with QoL status [33]. Participants who were 17 years old and above were found to have better QoL compared to younger participants, this is similar to other reports [49] and it may be a reflection of adaptation and experience that comes with age.

## Limitations of the study

Inferences could not be drawn to causal relationship among variables because of the cross-sectional nature of the study. Hearing loss was based on school’s report and self-report, no independent diagnostic assessment of the respondents’ hearing was done. Selection bias may be another possible limitation since only senior secondary students were recruited into the study. Though no Intelligent Quotient (IQ) test was conducted on the students before questionnaires were administered it may be safe to assume that senior secondary students may have a better IQ and able to easily fill out the questionnaire than junior secondary students. Moreover, the responses of the senior secondary students may reflect benefits of older age and experience. The WHOQOL-BREF questionnaire utilized in this study has not been validated for use among sign language users however, it was adopted for this study partly because its use had been demonstrated among DHH people in Nigeria [13]. Despite these limitations, this study provides unique insights into the QoL of DHH students in a developing country, which is useful in planning appropriate interventions.

## Conclusion

The poor QoL found in almost three-fifths of the study population shows the impact of hearing disability on the students’ QoL. This suggests that hearing loss imposes a serious challenge on the overall development of young people. The study found that the school type and socio-economic class have a relationship with the QoL of DHH students while age of onset of hearing loss and gender showed no significant relationship with QoL. The better QoL found among students in special schools in the environment and social relationship domains suggests that the Deaf community created in the special school provided a form of protection against stigma and discrimination, it also apparently promoted social interactions between DHH students.

## Supporting information

S1 Table Distribution of respondents by type of school. (DOCX)

S2 Table Social class of respondents according to type of school. (DOCX)

S3 Table QoL scores in various domains. (DOCX)

S4 Table Post hoc test results. (DOCX)
