# Supplementary material for: Quality of life of deaf and hard of hearing students in Ibadan metropolis, Nigeria
Source: PLoS One. 2018 Jan 2;13(1):e0190130. doi: 10.1371/journal.pone.0190130 (PMC5749760; doi:10.1371/journal.pone.0190130)
Supplement: S1 Table — (DOCX) [file pone.0190130.s001.docx]

**S1 Table: Distribution of respondents**

|  | **Name of school** | **Type of school** | **Number of respondents** | **Percentage (%)** |
| --- | --- | --- | --- | --- |
| **1** | Ibadan School for the Deaf | Total mainstream | 26 | 25.5 |
| **2** | IMG Oke-Ado | Total mainstream | 16 | 15.7 |
| **3** | Methodist Grammar School | Partial mainstream | 31 | 30.4 |
| **4** | Christian Mission Centre | Special | 29 | 28.1 |
|  |  | **Total** | **102** | **100** |

**Total mainstream: (n=42) Partial mainstream (n=31) Special (n=29)**
