# Supplementary material for: Quality of life of deaf and hard of hearing students in Ibadan metropolis, Nigeria
Source: PLoS One. 2018 Jan 2;13(1):e0190130. doi: 10.1371/journal.pone.0190130 (PMC5749760; doi:10.1371/journal.pone.0190130)
Supplement: S2 Table — (DOCX) [file pone.0190130.s002.docx]

**S2 Table:** **Social class of respondents**

| **Social class** | **Special**  **n (%)** | **Partial mainstream n(%)** | **Total mainstream n(%)** | **Total**  **n(%)** |
| --- | --- | --- | --- | --- |
| **Upper** | 21 (72.4) | 6 (19.4) | 2 (4) | 29(28.4%) |
| **Middle** | 6 (20.7) | 18 (58.1) | 20 (47.6) | 44(43.1%) |
| **Lower** | 2 (6.9) | 7 (22.5) | 20 (47.6) | 29(28.4%) |
| **Total** | 29 | 31 | 42 | 102 (100%) |
