# Supplementary material for: Quality of life of deaf and hard of hearing students in Ibadan metropolis, Nigeria
Source: PLoS One. 2018 Jan 2;13(1):e0190130. doi: 10.1371/journal.pone.0190130 (PMC5749760; doi:10.1371/journal.pone.0190130)
Supplement: S3 Table — (DOCX) [file pone.0190130.s003.docx]

**S3 Table: QoL Scores by Age, Age at onset of deafness, type of school attended, communication at home and parents’ hearing status.**

|  | **physical Mean±SD** | **Sig** | **psychological Mean±SD** | **Sig** | **social Mean±SD** | **Sig** | **enviroment Mean±SD** | **sig** |
| --- | --- | --- | --- | --- | --- | --- | --- | --- |
| **Age group** |  | 0.008* |  |  |  |  |  | 0.199 |
| 12-15 n= 15 | 11.09±2.17 |  | 11.56±2.80 |  | 10.76±2.11 |  | 10.83±2.55 |  |
| 16-19 n= 63 | 12.61±2.44 |  | 11.66±2.63 |  | 12.29±2.90 |  | 11.27±2.24 |  |
| 20-23 n= 20 | 13.71±2.17 |  | 12.83±2.47 |  | 11.67±2.11 |  | 11.55±2.29 |  |
| 24-27 n= 2 | 13.14±2.17 |  | 13.00±5.20 |  | 12.67±2.82 |  | 11.50±0.71 |  |
| 28 28-31 n= 2 | 16.00±3.23 |  | 10.33±2.36 | 0.373 | 13.33±1.89 | 0.290 | 15.00±0.01 |  |
| **Age group at onset of deafness** |  |  |  |  |  |  |  |  |
| 0-5 n=86 | 12.71±2.42 | 0.797 | 11.84±2.69 | 0.787 | 11.87±2.61 | 0.392 | 11.45±2.33 | 0.239 |
| >5 n=15 | 12.54±2.86 |  | 12.64±2.51 |  | 12.51±2.95 |  | 10.75±2.14 |  |
| **Type of school** |  |  |  |  |  |  |  |  |
| **Special n=29** | 13.39±2.21 |  | 12.14±2.77 |  | 12.96±3.04 |  | 12.60±2.34 |  |
| Partial mainstream n=31 | 12.33±2.23 |  | 11.20±2.22 |  | 11.82±2.28 |  | 10.93±1.95 |  |
| Total mainstream  n=42 | 12.40±4.49 | 0.182 | 11.78±2.75 | 0.182 | 11.39±2.5 | 0.046* | 10.76±2.23 | 0.002* |
| **Communication at home** |  | 0.878 |  | 0.535 |  | 0.186 |  | 0.618 |
| Lip reading n=8 | 12.29±2.55 |  | 10.92±2.71 |  | 12.17±2.51 |  | 10.87±2.17 |  |
| Writing n=36 | 12.65±2.21 |  | 11.83±2.68 |  | 12.59±2.75 |  | 11.14±2.63 |  |
| Sign language  n =58 | 12.75±2.66 |  | 12.03±2.63 |  | 11.56±2.58 |  | 11.52±2.12 |  |
| **Parent’s hearing status** |  |  |  |  |  |  |  |  |
| Deaf and deaf n=9 | 12.25±2.28 |  | 11.56±3.21 |  | 11.85±2.71 |  | 11.30±1.54 |  |
| One deaf n=3 | 13.33±2.57 |  | 12.21±3.85 |  | 11.56±4.30 |  | 12.00±2.29 |  |
| Both hearing n=90 | 12.70±2.51 | 0.789 | 11.90±2.65 | 0.912 | 12.01±2.63 | 0.951 | 11.31±2.38 |  |

(*indicates significant differences in the group, p ≤0.05)
