# Supplementary material for: Quality of life of deaf and hard of hearing students in Ibadan metropolis, Nigeria
Source: PLoS One. 2018 Jan 2;13(1):e0190130. doi: 10.1371/journal.pone.0190130 (PMC5749760; doi:10.1371/journal.pone.0190130)
Supplement: S4 Table — (DOCX) [file pone.0190130.s004.docx]

**S4 Table: Post hoc test results of the social and environment domains of WHOQOLBREF across the deaf schools**

|  | **Type of School** | | **Sig** |  |
| --- | --- | --- | --- | --- |
| **Physical** |  |  |  |  |
| **Psychological** |  |  |  |  |
| **Social** | Special | Partial mainstream | 0.214 | Df (2,99)  F= 3.182 |
|  |  | Total Mainstream | 0.038* |  |
|  | Partial mainstream | Total mainstream | 0.765 |  |
| **Environment** | Special | Partial mainstream | 0.011* | Df (2,99)  F= 6.863 |
|  |  | Total Mainstream | 0.002* |  |
|  | Partial mainstream | Total mainstream | 0.946 |  |

(*indicates significant post hoc test between the different type of deaf schools, p ≤0.05)
